# Can we use the tools we already have to help patients in need? Evaluating practice-based evidence of analgesic effects from intermittent theta burst stimulation for treatment of depression

**DOI:** 10.1080/24740527.2024.2310806

**Published:** 2024-01-29

**Authors:** P. Maxwell Slepian

**Affiliations:** Transitional Pain Service, Toronto General Hospital, University Health Network, Toronto, Ontario, Canada; Department of Anesthesia and Pain Management, Toronto General Hospital, University Health Network, Toronto, Ontario, Canada; Krembil Research Institute; Department of Anesthesiology and Pain Medicine, University of Toronto, Toronto, Ontario, Canada; Department of Psychology, York University, Toronto, Ontario, Canada

It is well established that pain and mental health disorders, and in particular depression, are highly comorbid.^1^ Although prevalence estimates vary widely, the odds of an individual with chronic pain also being diagnosed with depression are approximately 1.4–4 times greater than that of the general population.^1^ The magnitude of this issue cannot be understated. In a recent study using the U.S. population-based National Health and Nutrition Examination Survey, 4.4% of respondents reported both chronic pain and depression.^2^ This suggests that nearly 15 million Americans have comorbid chronic pain and depression, and that proportion is very likely to be similar in Canada. Moreover, there is a bidirectional relationship between pain and depressive symptoms, such that they mutually maintain and exacerbate each other.^3^ Despite widespread acknowledgment of these relationships, dual targeted treatments are limited and new treatment approaches are sorely needed.^4^

Noninvasive brain stimulation, particularly transcranial magnetic stimulation (TMS) and its extension repetitive TMS (rTMS), has been forwarded as a potential treatment strategy that can target both chronic pain and depression.^5,6^ TMS uses magnetic pulses to alter neural activity in the cortex corresponding to the location of a magnetic coil over the scalp. Variations in stimulation protocol, including pulse duration, frequency, and intensity, as well as stimulation location can lead to varying outcomes. In general, low-frequency stimulation (1 Hz) inhibits neural activity, whereas high-frequency stimulation (>5 Hz) facilitates neural activity. TMS was first developed to study simple effects of single-pulse stimulation. However, rTMS creates a lasting effect believed to be caused by a long-term potentiation or depression of neurons being stimulated.^7,8^ A single session of rTMS, generally lasting approximately 40 min with repeated pulses delivered at 120% of motor threshold (i.e., 120% of the intensity of stimulation required to cause muscle activation in the limb), has been found to have analgesic effects.^8^ Meta-analytic evidence supports the use of repeated rTMS sessions (approximately 40 sessions) over the primary motor cortex (M1), but not over the dorsolateral prefrontal cortex (DLPFC), for treatment of pain.^9^ In contrast, rTMS protocols targeting the DLPFC are efficacious for treatment of depression and are particularly recommended for treatment-resistant depression.^10,11^ Indeed, such protocols have regulatory approval and are funded in Canada.

Intermittent theta-burst stimulation (iTBS) is a relatively newer rTMS stimulation protocol that uses a lower stimulation intensity (i.e., 80% of motor threshold) delivered at a higher frequency, matching the brain’s natural theta brain wave rhythm.^7^ This stimulation pattern is more effective at inducing lasting long-term potentiation/depression effects, requiring approximately 10% of the active stimulation required to incur the same physiological effect as standard rTMS protocols.^7,12^ Although preliminary evidence for iTBS analgesia is promising, studies examining this have primarily focused on M1 stimulation and treatment of neuropathic pain.^13,14^ However, both unilateral left-sided and bilateral DLPFC stimulation with iTBS have been found to be noninferior to standard high-frequency rTMS for treatment of depression.^15,16^ Consequently, iTBS protocols are currently approved and already in use for the treatment of depression. It was in this context that Kirupaharan and colleagues conducted a retrospective chart review of patients undergoing bilateral iTBS of the DLPFC for treatment of depression.^17^

Kirupaharan and colleagues reviewed data from 104 patients undergoing iTBS of the bilateral DLPFC for treatment of depression, with either 25 daily sessions (*n* = 73) or 30 twice-daily sessions (*n* = 31).^17^ As might be expected based on the high comorbidity between depression and pain, half of these patients reported moderate pain before their first treatment session, with 22 patients reporting a chronic pain diagnosis (unspecified, *n* = 10; fibromyalgia, *n* = 6; chronic back pain, *n* = 4; chronic migraine, *n* = 1; chronic temporomandibular joint pain, *n* = 1).^17^ Patients reported their pain intensity on a visual analogue scale (VAS) before treatment and at the completion of sessions 6, 11, 16, and 21 and after treatment conclusion. Reported pain intensity reduced from before to after treatment for the 32 patients who completed both pre- and posttreatment VAS.^17^ Seventeen of these participants started with moderate pain (VAS > 40), and this subgroup showed the same result. About half of the patients who had improvement in pain during iTBS also had improvement in depressive symptoms.^17^

Though this evidence appears promising, there are a number of notable limitations that muddy the interpretation of these data. This was a retrospective study of observational data that were not collected for the purpose of research. Thus, it is subject to all of the standard threats to internal validity that may preclude drawing strong conclusions. In particular, it is entirely possible that patients simply regressed to their mean pain throughout treatment. There was no obvious evidence of attrition bias on inspection, but that also does not mean that data were missing completely at random. The absence of data on pain or medical history for the majority of participants and only very limited information for the remainder is another critical limitation.^17^ Less than half of the patients reporting pain reported having chronic pain, and only a portion of those provided information about their pain and analgesic use.^17^ It is possible that patients were experiencing acute injury that healed over the course of treatment. It is also possible that patients began other therapies for pain during the iTBS protocol. Perhaps most notable, and not acknowledged by the authors, is an issue that arises from when pain was measured in relation to the TMS sessions. Pain was rated before TMS for the first session and only directly after TMS in subsequent visits.^17^ It is possible that the pre- versus posttreatment comparison is just capturing an acute analgesic effect of the TMS stimulation and not a cumulative effect as the authors imply.

Despite these reservations, the report by Kirupaharan and colleagues is a meaningful, if *very* preliminary, practice-based evaluation of iTBS for pain intensity among individuals undergoing treatment for depression. Although only a small group of patients provided full information, and only some of those had improvement in pain (31.2% with greater than 30% reduction in pain) and only a subset of those experienced improvement in both pain and depressive symptoms,^17^ any treatment associated with concurrent improvement in pain and depressive symptoms is promising. Several randomized controlled trials testing iTBS for concurrent treatment of pain and depression are underway, including one comparing iTBS of the DLPFC, iTBS of M1, and simultaneous iTBS of the DLPFC and M1 for treatment of comorbid pain and depression (clinicaltrials.gov registration no. NCT04556890). However, trial results will not be reported for several more years. In the meantime, iTBS for depression has regulatory approval and is already in regular use. Evaluating available practice-based data is a first step to making it routinely available to patients with comorbid pain and depression. Although it is not possible to draw strong conclusions from the paper by Kirupaharan et al., it does encourage collection and reporting of pain-related data in settings delivering rTMS for depression treatment. Robust collection of safety data, including adverse events, side effects, and patient experiences, is also needed. By bolstering measurement practices in clinical care, we can improve flexibility to offer such treatments “off-label” for comorbid pain and depression.
